# Identification and characterization of the pathogenic potential of phenol-soluble modulin toxins in the mouse commensal *Staphylococcus xylosus*

**DOI:** 10.3389/fimmu.2022.999201

**Published:** 2022-09-15

**Authors:** Kunal Reshamwala, Gordon Y. C. Cheung, Roger C. Hsieh, Ryan Liu, Hwang-Soo Joo, Yue Zheng, Justin S. Bae, Thuan H. Nguyen, Amer E. Villaruz, Alfonso S. Gozalo, William R. Elkins, Michael Otto

**Affiliations:** ^1^ Pathogen Molecular Genetics Section, Laboratory of Bacteriology, National Institute of Allergy and Infectious Diseases (NIAID), United States (US) National Institutes of Health (NIH), Bethesda, MD, United States; ^2^ Comparative Medicine Branch (CMB), NIAID, NIH, Bethesda, MD, United States

**Keywords:** *Staphylococcus xylosus*, *Staphylococcus aureus*, phenol-soluble modulin, delta-toxin, virulence, cytolysin, atopic dermatitis

## Abstract

In contrast to the virulent human skin commensal *Staphylococcus aureus*, which secretes a plethora of toxins, other staphylococci have much reduced virulence. In these species, commonly the only toxins are those of the phenol-soluble modulin (PSM) family. PSMs are species-specific and have only been characterized in a limited number of species. *S. xylosus* is a usually innocuous commensal on the skin of mice and other mammals. Prompted by reports on the involvement of PSMs in atopic dermatitis (AD) and the isolation of *S. xylosus* from mice with AD-like symptoms, we here identified and characterized PSMs of *S. xylosus* with a focus on a potential involvement in AD phenotypes. We found that most clinical *S. xylosus* strains produce two PSMs, one of the shorter α- and one of the longer β-type, which were responsible for almost the entire lytic and pro-inflammatory capacities of *S. xylosus*. Importantly, PSMα of *S. xylosus* caused lysis and degranulation of mast cells at degrees higher than that of *S. aureus* δ-toxin, the main PSM previously associated with AD. However, *S. xylosus* did not produce significant AD symptoms in wild-type mice as opposed to *S. aureus*, indicating that promotion of AD by *S. xylosus* likely requires a predisposed host. Our study indicates that non-specific cytolytic potency rather than specific interaction underlies PSM-mediated mast cell degranulation and suggest that the previously reported exceptional potency of δ-toxin of *S. aureus* is due to its high-level production. Furthermore, they suggest that species that produce cytolytic PSMs, such as *S. xylosus*, all have the capacity to promote AD, but a high combined level of PSM cytolytic potency is required to cause AD in a non-predisposed host.

## Introduction

Atopic dermatitis (AD) is an inflammatory skin disease that predominantly affects children and generally becomes less severe upon reaching adulthood (1). The pathophysiology of AD is a complex process, driven by environmental and genetic factors, barrier dysfunction, alterations in cell-mediated immune responses (e.g. pronounced Th2 immune responses in acute AD), and IgE-mediated hypersensitivity (2). Clinically, patients are presented with intense pruritus, chronic eczematous skin lesions, and epidermal thickening and hypertrophy (3). The inflammatory reactions in the epidermis are initiated by specialized antigen-presenting cells, such as Langerhans cells (4), which activate keratinocytes to release potent inflammatory mediators (4–7). These in turn (i) stimulate the expression of adhesion molecules in cells of the endothelium, thus attracting migration of macrophages and eosinophils to the site of inflammation (8), (ii) help induce a Th2-inflammatory phenotype (3, 9), and (iii) promote class-switching of B cells to produce Immunoglobulin E (IgE) (10, 11). Antigen-specific IgE molecules are recognized by the high-affinity receptor, FcϵRI, found on eosinophils, basophils and mast cells (12, 13) and upon their engagement of surface-bound IgE to multivalent antigens, many pro-inflammatory cellular processes are activated, including the rapid degranulation and release of preformed inflammatory mediators stored in intracellular granules (14), as well as the *de novo* synthesis and release of more pro-inflammatory mediators (15, 16). Mast cells are generally regarded as the main effector cell involved in that response (17).

While much is known about the mechanisms leading to skin inflammation in AD, we still do not have a complete understanding of the pathogenesis of AD, especially in the context of microbial infiltration in the skin. *Staphylococcus aureus* is a human pathogen associated with AD and AD severity (18, 19). More recently, a defined microbial factor produced by *S. aureus* could be linked to AD pathogenesis (20). This factor, δ-toxin, is a 26 amino-acid peptide toxin belonging to the family of phenol-soluble modulin (PSM) peptides (21, 22). PSMs are peptides of ~ 2 kDa (α-type) or 4 – 5 kDa (β-type) with cytotoxic and pro-inflammatory activities. They all contain amphiphilic α-helices, which give them a surfactant-like character that likely is responsible for their non-specific membrane-disturbing impact on a variety of cell types, leading to their lysis. Most staphylococci produce a species-specific repertoire of PSMs, frequently with only moderate or even lacking amino acid sequence similarity to those of other species. In many staphylococcal species other than *S. aureus*, PSMs are virtually the only secreted toxins. While in the initial study on the role of δ-toxin in AD (20), promotion of AD was driven exclusively by δ-toxin, a further study more generally implicated PSMs in mast cell degranulation as a hallmark of AD development (23). The reason for a potentially specific involvement of δ-toxin as opposed to other PSMs, which all share similar biological activities, has remained unknown.

*Staphylococcus xylosus* is a commensal of mice and other mammals, but rarely humans, where - like most other staphylococcal species that colonize mammals - it usually does not cause any harm (24). However, there are reports on skin infections in mice that are caused by *S. xylosus* (25, 26), in particular skin lesions with AD-like symptoms (27–31). We hypothesized that *S. xylosus* produces PSMs with pathogenic potential that may also trigger AD pathogenesis. Therefore, we here identified and characterized PSMs of *S. xylosus*, produced isogenic deletion mutants in the main detected *psm* genes, and used pure PSMs as well as supernatants of cultures of *S. xylosus* and its isogenic *psm* mutants to investigate the pathogenic potential of *S. xylosus* PSMs with a focus on AD pathogenesis. Our results not only give insight into what are most likely the main virulence determinants of *S. xylosus*, but give important general insight into the role of δ-toxin and PSMs in AD. Notably, they help explain why δ-toxin has an exceptional role in promoting AD.

## Materials and methods

### Ethics statement

Animal experiments and protocols were performed according to the regulations of the Division of Intramural Research Animal Care and Use Committee (DIR ACUC) of the National Institute of Allergy and Infectious Diseases (NIAID), animal study proposal LB1E. Animal work was conducted adhering to the institution’s guidelines for animal use and followed the guidelines and basic principles as outlined in the United States Public Health Service Policy on Humane Care and Use of Laboratory Animals, and the Guide for the Care and Use of Laboratory Animals by certified staff in an Association for Assessment and Accreditation of Laboratory Animal Care (AAALAC) international accredited facility. All animals were euthanized with CO_2_ at the end of the studies. Human neutrophils were isolated from venous blood of healthy volunteers in accordance with a protocol (No. 99-CC-0168) approved by the Institutional Review Board for Human Subjects, NIAID. Informed written consent was obtained from all volunteers.

### Animals

Female 10- to 12-week-old SKH-1E mice (Charles River Laboratories) were used for mouse colonization/infection studies and C57BL/NCrl mice (Charles River Laboratories) were used for Evans blue sensitization experiments and for the collection of bone marrow for neutrophil and mast cell differentiation. All mice were bred and maintained under pathogen-free conditions in an AAALAC-accredited animal facility in the NIAID. Age-, sex- and littermate-matched mice were randomly assigned into treatment groups in each experiment.

### Bacterial strains and constructs and growth conditions

The *S. xylosus* strains used in this study comprised of reference laboratory strains and a series of strains from a collection of skin isolates obtained from C57BL/6 mice [wild-type (WT) or associated genetically altered strains] with spontaneous ulcerative dermatitis at the NIH **(** **Supplementary Table S1** **)**. For the identification of *S. xylosus* PSMs from culture filtrates, all isolates and reference strains from glycerol stocks were first streaked onto tryptic soy agar plates and incubated overnight at 37 °C. The following day, a single clone was inoculated into tryptic soy broth (TSB) and grown overnight with shaking at 37 °C and 180 rpm. Overnight cultures were then inoculated at 1:100 dilution into TSB and grown for 8 hours. At these time points, culture supernatants were collected and stored at -20 °C until needed. For *in vitro* experiments with culture filtrates, supernatant from 16-hour cultures were passed through 0.22-μm polyethersulphone (PES) membrane filters (Sigma-Aldrich).

### Construction of isogenic gene deletion mutants in *S. xylosus*

To create markerless isogenic *psm* mutants in *S. xylosus* AG14, an allelic replacement procedure was employed (32). PCR primer pairs **(** **Supplementary Table S2** **)** were designed to amplify ~ 1-kb regions upstream and downstream of the *psm*α and *psm*β1 genes in *S. xylosus* AG14 genomic DNA **(** **Supplementary Table S2** **).** Overlap PCR was performed with the upstream and downstream ~ 1-kb PCR fragments with primers Xylpsmα1 and Xylpsmα2 (Reverse) and Xylpsmβ1 and Xylpsmβ2 (Reverse) to create ~ 2 kb fragments, which lack the coding sequences for *psm*α and *psm*β1, respectively, and then cloned into the SmaI restriction site of plasmid pIMAY (32) resulting in pIMAY-Δ*psm*α and pIMAY-Δ*psm*β1. Plasmids were first transformed into *S. carnosus* TM300 and then into *S. aureus* PS187ΔΔ. Finally, the plasmids were transduced into *S. xylosus* AG14 with phage φ187 as described previously (33) and the allelic replacement procedure was performed essentially as described (32, 33) to create the single *psm*α and *psm*β1 mutants. To create the double *psm*α and *psm*β1 mutant, pIMAY-Δ*psm*β1 was phage-transduced into the Δ*psm*α mutant, followed by allelic replacement. The fidelity of all constructs was confirmed by DNA sequencing and reversed-phase high pressure liquid chromatography/electrospray ionization mass spectrometry (RP-HPLC/ESI-MS) of culture filtrates.

### N-terminal sequencing

PSMα of *S. xylosus* was subjected to N-terminal Edman sequencing performed by the Research Technology Branch of NIAID after boiling in 25% trifluoroacetic acid (TFA) for 2 h at 55°C for removal of potentially existing N-terminal N-formyl modification (34).

### PSM detection and quantification

PSM production by *S. xylosus* strains was analyzed using a SOURCE 15RPC ST 4.6/100 column (Cytiva) using a linear gradient from 0.1% TFA in water to 0.1% TFA in acetonitrile over ~ 20 column volumes at a flow rate of 0.5 ml/min on an Agilent 1100 HPLC system connected to an Agilent Quadrupole LC/MS instrument with an ESI source. For purification of PSMα, we used a Zorbax SB-C18 5 μm 9.4 × 250 mm column (Agilent) and a GE AKTA Purifier system using the same buffers and a linear gradient from 0 to 100% buffer B in 20 column volumes. Further analysis of PSM production in *S. xylosus* strains was performed using our routine RP-HPLC/ESI-MS analysis method as for *S. aureus* or *S. epidermidis* by adapting the m/z values (two per PSM) for *S. xylosus* PSMs (35). This method was also used for the absolute quantification of PSMs in culture filtrates with standard curves obtained using known molar concentrations of synthetic PSMs.

### Synthetic peptides

All PSM peptides were synthesized with an N-terminal N-formyl methionine modification at > 95% purity by Peptide 2.0: *S. aureus* PSMα3 (MEFVAKLFKFFKDLLGKFLGNN); *S. aureus* δ-toxin (MAQDIISTIGDLVKWIIDTVNKFTKK); *S. xylosus* PSMα (MSFIIDIIKKIVGLFKGE), *S. xylosus* PSMβ1 (MAEIVEAIGKAVSAGLSHDWATMGVSIAEVLGKGVDFVLGFFK). Peptides were dissolved in DMSO and stored at -20 °C.

### Isolation of neutrophils from mouse bone marrow

The isolation of neutrophils from mouse bone marrow was performed as described previously (36). Briefly, 8- to 10-week-old C57Bl/6NCrl female mice were sacrificed followed by the collection of the tibiae and femora. The bones were flushed with phosphate-buffered saline (PBS) with 1% (v/v) bovine serum albumin (BSA) and the bone marrow was sedimented after brief centrifugation. The erythrocytes in the cell pellet were lysed after brief exposure to ACK lysis buffer (Lonza), and magnetic negative selection was used to purify neutrophils, which were finally resuspended in RPMI-1640 media without phenol red (Gibco) and 50 mM 4-(2-hydroxyethyl)-1-piperazineethanesulfonic acid (HEPES) buffer (Invitrogen) [RPMI-H], to the desired concentration.

### Isolation of mouse bone marrow-derived mast cells

The isolation of mouse bone marrow mast cells (mBMMCs) was performed as described previously (37). Briefly, after sedimentation of the erythrocyte-depleted bone marrow with ACK lysis buffer, the cells were resuspended in 10 ml of RPMI-1640 media (Gibco), supplemented with 1% (v/v) Penicillin-streptomycin-glutamine (Invitrogen), 2.5% (v/v) HEPES (Invitrogen), 1% (v/v) Non-essential amino acids (Invitrogen), 100 mM sodium pyruvate (Invitrogen), 50 mM β-mercaptoethanol (Sigma-Aldrich) and 10% (v/v) fetal bovine serum (Gibco) and supplemented with a final concentration of 0.4 μg/ml each of murine recombinant IL-3 (m-IL-3) and Stem Cell Factor (SCF) (PeproTech) at a final concentration of ~ 5 × 10^5^ cells/ml. Approximately every 1-2 days for the first week, non-adherent cells were transferred to a new culturing vessel and diluted two-fold if a concentration of 1 × 10^6^ cells/ml was reached. Thereafter, cells were diluted once every week with fresh media supplemented with m-IL-3 and SCF. After 3-4 weeks of propagation, the presence of mast cells was confirmed by flow cytometric analyses for the presence of both CD117 (c-Kit) and FcϵR surface markers.

### Isolation of human neutrophils

Human neutrophils were isolated from heparinized blood from healthy donors as described previously (38). Briefly, erythrocytes and peripheral blood mononuclear cells were removed with Dextran (Sigma-Aldrich) and Ficoll-Paque PLUS (GE Healthcare), respectively. A final lysis step with sterile water removed contaminating erythrocytes and the resulting cell pellet of >90% neutrophils was resuspended in RPMI-H and enumerated by microscopy.

### Cell lines

The human mast cell line LAD2 was derived from CD34^+^ cells following marrow aspiration of a patient with aggressive mastocytosis (39). These cells were cultured in StemPro-34 (Life technologies) supplemented with 2% (v/v) StemPro nutrient supplement (Life technologies), Penicillin-streptomycin-L-glutamine (Life technologies), and 100 ng/ml recombinant human stem cell factor (rhSCF) (Peprotech). Cells were diluted two-fold after a concentration of ~ 5 × 10^5^ cells/ml was reached. Mast cell purity was confirmed by routine flow cytometric analyses for the presence of both CD117 (c-Kit) and FcϵR surface markers.

### Measurements of erythrocyte lysis (hemolysis)

Whole blood from anesthetized mice was collected by a cardiac puncture procedure. After collection, bleeds were immediately transferred into heparinized tubes (Sarstedt), stored at 4°C and used within two days (as with freshly collected heparinized human blood). Hemolysis was measured by incubating 100 μl of synthetic PSMs or culture supernatants with 100 μl of 4% (v/v) washed erythrocytes for one hour at 37°C in 96 well tissue culture plates, as previously described (40). The levels of released hemoglobin were determined by scanning at OD_540nm_ using a TECAN^®^ Spark microplate reader. Next, the percentage of lysis was calculated by first subtracting readings from media-alone blanks from all test readings from and then by dividing the absorbance values of the test wells by those obtained with a Triton X-100 (Sigma-Aldrich) positive control indicating 100% lysis.

### Real-time calcium imaging

Calcium flux in murine or human neutrophils was measured as previously described (36). Briefly, neutrophils were suspended at a concentration of 5 × 10^6^ cells/ml in RPMI/H and incubated with ~ 2 mM Fluo3-AM (Invitrogen, F1242) for 20 minutes at 37°C. Cells were then washed three times with HEPES buffer, resuspended to a concentration of 5 × 10^5^ cells/ml and dispensed into wells of a black 96-well plate (Costar) in 200-ml volumes. The plate was centrifuged at 240 × g for 5 minutes and calcium fluxes were monitored in a Spark^®^ Multimode Microplate Reader (Tecan) by measuring fluorescence using excitation and emission wavelengths of 485 nm and 535 nm, respectively. Fluorescence signals from the non-stimulated controls were subtracted from stimulated samples to account for background.

### Measurement of β-hexosaminidase release by mast cells

The detection of β-hexosaminidase from mBMMCs and human LAD2 cells was performed using p-nitrophenyl N-acetyl-β-D-glucosamide (PNAG) as previously described (41). Briefly, cells were grown in their corresponding media for six hours without IL-3 or SCF. mBMMCs and LAD2 cells were then sensitized overnight with mouse monoclonal anti-dinitrophenyl IgE (Sigma-Aldrich) and biotinylated anti human IgE (SeraCare), respectively. The following day, the cells were washed three times with sterile HEPES buffer (10 mM HEPES, Invitrogen), 137 mM sodium chloride (Sigma-Aldrich), 2.7 mM potassium chloride (Sigma-Aldrich), 0.4 mM sodium phosphate dibasic (Fisher), 5.6 mM glucose (Macron), 1.8 mM calcium chloride (Sigma-Aldrich), 1.3 mM magnesium sulfate (Sigma-Aldrich), 0.04% (w/v) bovine serum albumin, pH 7.4) and resuspended to concentrations at 1 × 10^6^ cells/ml (LAD2 cells) or 5 × 10^6^ cells/ml (mBMMCs). To wells of a 96 well plate, 90 μl of cells were added and first allowed to equilibrate at 37°C for 30 minutes prior to the addition of 10-μl samples. After co-incubation at 37°C for 20 minutes, plates were centrifuged and samples from each well were collected for LDH release (see below) and β-hexosaminidase release. Briefly, for the latter, plates were first centrifuged at 450 × g, at 4°C for 5 min and 50 μl of supernatant from each well was transferred to a new flat-bottomed 96 well plate. To the remaining cells, 50 μl of 1% (v/v) Triton X-100 was added to each well to yield 100% lysis and then 50 μl of each lysate was transferred to a new plate. Then, to all wells, 100 μl of PNAG (3.5 μg/ml) in citrate buffer (40 mM citric acid (Sigma-Aldrich), 20 mM sodium phosphate dibasic (Fisher, pH 4.5) was added and plates were incubated for 90 min at 37°C. The reactions were stopped with 50 μl of 400 mM glycine (pH 10.7, Sigma-Aldrich). Absorbances at 405_nm_ and 620_nm_ (background) were measured using a TECAN^®^ Spark multiplate reader. The percentage of β-hexosaminidase released was determined by subtracting the 620_nm_ values from the 405_nm_ values and then dividing the supernatant values by 100% lysis values from the same well.

### Lysis measurements of neutrophils and mast cells

For both human and mouse neutrophils, 100 μl of cells at a concentration of 2.5 × 10^5^ cells per ml were incubated with 100 μl of sample for one hour at 37°C (20 min at 37°C for mast cells – see above) in 96 well tissue culture plates. Cell lysis was determined using a Cytotoxicity Detection Kit (Roche Applied Sciences) by release of lactate dehydrogenase (LDH), as described previously (38), and absorbance readings recorded in a TECAN^®^ Spark microplate reader. After accounting for background by subtracting values of blanks, the percentage of lysis of each cell type was determined by dividing the absorbance values of the test wells by those obtained with a 1% (v/v) Triton X-100 (Sigma-Aldrich) positive control representing 100% lysis.

### Mouse AD model

For animal infections, overnight cultures of bacterial strains were inoculated into TSB at 1:100 and allowed to grow for ~ 3 h at 37°C with shaking at 180 rpm. Bacteria were washed with sterile PBS and then adjusted to the desired CFU concentration. The murine AD model was performed essentially as previously described (20), with modifications. Briefly, 10- to 12-week-old SKH-1E female mice (Charles River Laboratories) were anesthetized and the back skin sterilized with an alcohol swab. A 1 cm × 1 cm piece of sterile gauze was placed on the dorsal area followed by the application of a 100-μl bacterial suspension containing 1 × 10^8^ CFUs. Mice dorsa were bandaged with transparent occlusive dressing (Tegaderm™, 3M) to sustain bacterial contact with the skin. After seven days, mice were euthanized by CO_2_ and the occlusive dressing and gauze were removed. A 1 cm × 1 cm piece of the dorsal skin was excised in the area of gauze-skin contact and then subjected to histopathological analysis with hematoxylin and eosin (H and E) staining. The slides were examined by a veterinary pathologist and a score of inflammation ranging from 0 – 4 was used, with 4 indicating the greatest degree of inflammation.

### Protein-fragment complementation assay

To assess mast cell degranulation *in vivo*, the PCA assay was performed as described previously (42) with the following modifications. Briefly, the ears of wild-type C57Bl/6NCrl mice were sensitized by intradermal injections of 20 ng of α-DNP-IgE (Sigma-Aldrich) resuspended in saline. After 15 hours of incubation, the mice were intradermally challenged with 20 μl containing 2% culture supernatants. Following inoculation, 100 μl of 5 mg/ml Evans blue dye (Sigma-Aldrich), diluted in saline, was intravenously injected into each mouse. After 30 minutes, the mice were sacrificed, and a biopsy was collected using a 5-mm diameter punch (Integra) from each ear. The punches were then placed in 200 μl formamide (Sigma-Aldrich) solution and incubated at 63°C overnight with shaking. Following this incubation, 150-μl formamide extracts were dispensed into wells of a clear flat-bottomed polypropylene 96 well plate and the absorbance values were measured at 600_nm_.

### Statistical analysis

Graph Pad Prism (version 9.1.1) was used for statistical analyses. Unpaired, two-tailed Student’s t-tests were used when comparing two groups, and one-way or two-way ANOVA with Tukey’s post-tests when comparing more than two groups. ANOVAs were only used when data passed normality tests (Anderson–Darling, D’Agostino–Pearson, Shapiro–Wilk, Kolmogorov–Smirnov). Otherwise, non-parametric tests were used. All error bars depict the standard deviation.

## Results

### Identification of *S. xylosus* PSMs

**Figure 1:**
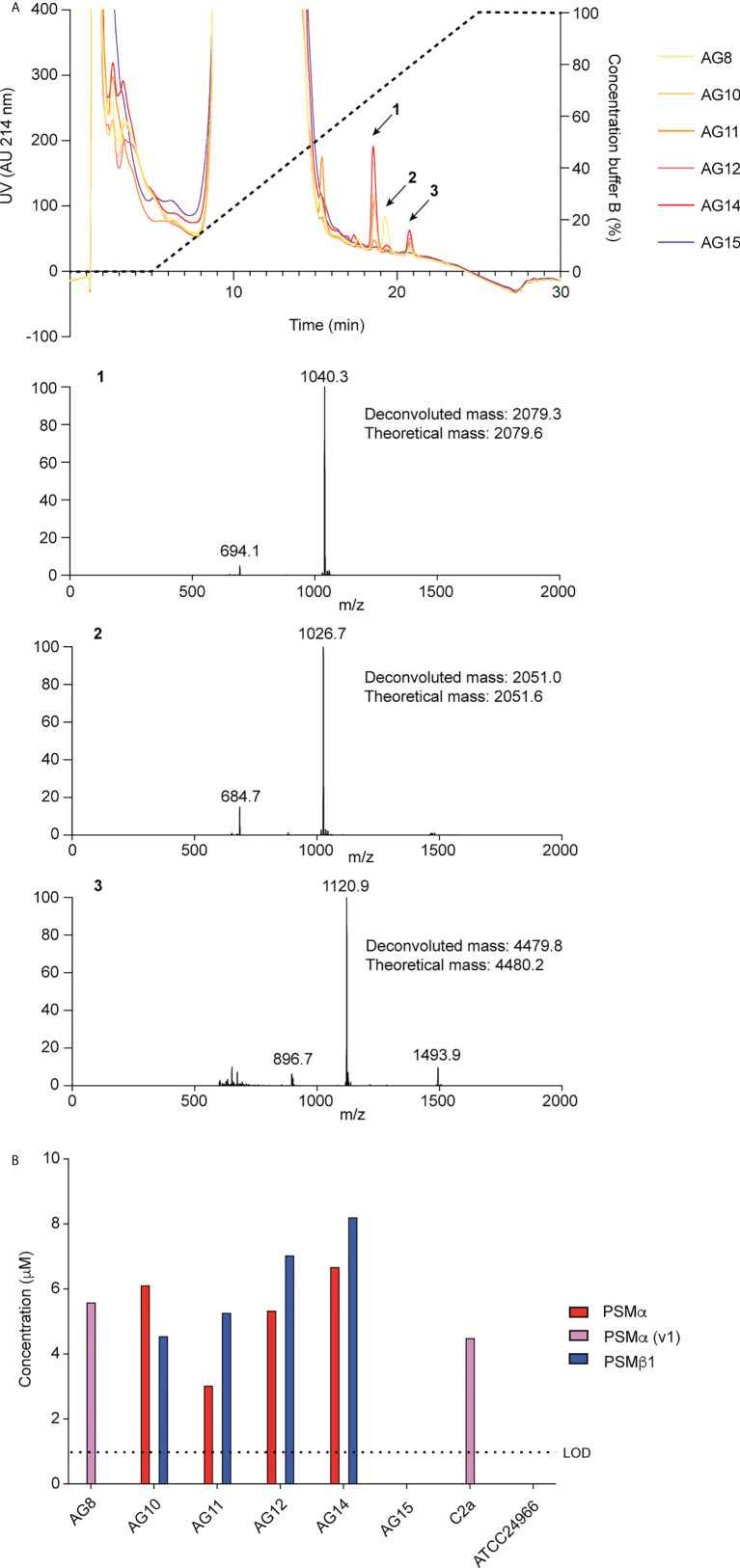
Detection of *S. xylosus* PSMs. **(A)** RP-HPLC/ESI-MS of culture filtrates of *S. xylosus* clinical isolates. Extracted mass spectra of peaks 1 to 3 are shown below the chromatogram. Results from deconvolution and theoretical average isotopic masses are noted. **(B)** Analysis of PSM production pattern by RP-HPLC/ESI-MS of culture filtrates collected from clinical *S. xylosus* isolates and reference strains. LOD, limit of detection.

To identify PSMs of *S. xylosus*, we used *S. xylosus* isolates obtained from spontaneous ulcerative dermatitis in mice and RP-HPLC/ESI-MS. In the characteristic elution range of PSMs, which elute exceptionally late during RP-HPLC due to their amphiphilic nature (21), we found three predominant peaks with deconvoluted masses of 2079.3 Da (peak 1), 2051.0 Da (peak 2), and 4479.8 Da (peak 3) in this order of elution (**Figure 1A**). Four out of six clinical isolates showed a similar pattern of production of peaks 1 and 3, one isolate only produced peak 2, and one isolate did not reveal PSM production. We also measured production of the peptides associated with these peaks in two *S. xylosus* standard strains, one of which showed a pattern similar to the clinical isolates with production of only peak 2 (strain C2a), and the other did not reveal production of PSM peptides (strain ATCC 24966) (**Figure 1B**).

PSMs can be classified in α-type PSMs, which have molecular weights (MWs) of ~ 2 kDa, and β-type PSMs, which have MWs of 4 to 5 kDa (22). Due to their higher MWs and similarity to known PSMs, β-type PSMs are usually annotated as such, or as hemolytic peptides, in staphylococcal genomes. In contrast, the identity of α -type PSMs cannot be deduced from genome sequences, as their length is below common annotation thresholds for open reading frames. Thus, the production of α-type PSMs in species where PSMs have not yet been characterized requires purification and N-terminal sequence conformation by Edman sequencing, in addition to the MW analysis provided by RP-HPLC/ESI-MS.

**Figure 2:**
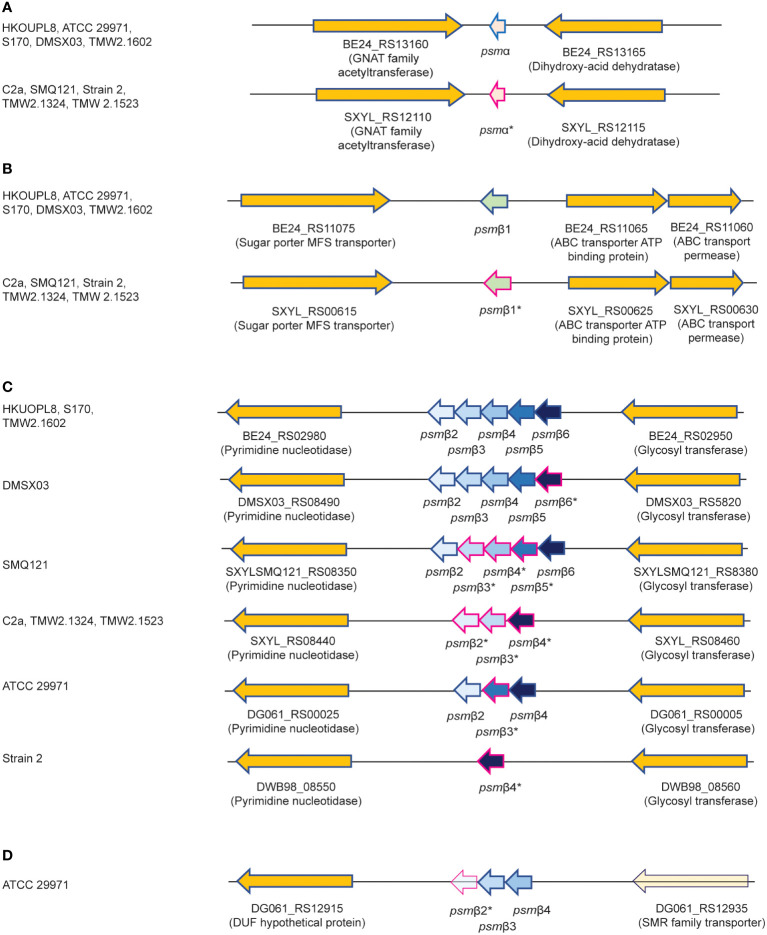
Amino acid sequences of *S. xylosus* PSMs and encoding genetic loci. **(A)** Genetic location of the *psm*α gene. **(B)** Genetic location of the *psm*β1 gene. **(C)** Genetic location of the *psm*β2-6 operon. Strain HKUOPL8 is used for reference (underlined) for the location of *psm*α, *psm*β1 and *psm*β2-6. **(D)** Genetic location of a second *psm*β operon in ATCC 29971. *Genes encoding PSM variants are denoted by a fuchsia border around the arrow depicting the gene. For some PSMβ-type peptides, multiple variants exist but are not distinguished here. A complete list of predicted amino acid sequences of *S. xylosus psm* genes and variants can be found in **Table 1**.

We therefore purified the main α-type PSM (peak 1) by RP-HPLC on a semi-preparative column. We assumed that the smaller α-type PSM peak 2 corresponded to an N-formylated version of peak 1, owing to their mass difference of 28 Da. PSMs are secreted as the primary translation product by a dedicated transporter (43), thus carrying an N-terminal N-formyl methionine like all primary bacterial translation products. However, some peptide molecules can undergo N-terminal deformylation by peptide deformylase (44), leading to a mass difference of 28 Da. Pairs of the same PSM peptides with a mass difference of 28 Da are therefore commonly found in PSM analyses and we thus assumed first that peaks 1 and 2 may represent N-formylated and N-deformylated versions of the same PSM peptide. N-terminal sequencing of the purified peptide corresponding to peak 1 yielded a sequence of MSFIIDIIKKIVGLFKGE, which represents the entire peptide and is in perfect agreement with the detected MW. The MW as determined by HPLC/MS and N-terminal sequence information, together with analysis of available *S. xylosus* genome sequences identified peak 1 as the peptide product of a gene situated between those encoding a GNAT family acetyltransferase and a dihydroxy-acid dehydratase in several *S. xylosus* genomes (**Figure 2A**). Following the nomenclature we established previously for staphylococcal PSMs (21), we called this peptide PSMα of *S. xylosus*.

We then analyzed all available *S. xylosus* genome sequences for sequences with similarity to that α-type peptide and for genes annotated as encoding PSMβ-like peptides, as well as sequences with similarity to PSMβ peptides found by this procedure. Results are shown in **Figure 2** and deduced amino acid sequences in **Table 1**. Notably, we found that in several *S. xylosus* genomes a variant of the *psm*α gene is present, which encodes a peptide with a mass difference of 28 Da as compared to PSMα. As this peptide variant is found in the genome of strain C2a, where we also found peak 2 (rather than peak 1) using RP-HPLC/ESI-MS, together with the facts that (i) only PSMα (peak 1) or its variant (peak 2) but not both appear to be produced by different isolates, and (ii) N-formylated versions of the same PSM commonly elute later, not earlier, during RP-HPLC than N-deformylated forms, we concluded that peaks 1 and 2 stem from two different peptides, PSMα and PSMα (v1), whose mass difference only coincidentally is the same as that due to N-terminal formylation.

**Table 1:** Amino acid sequences of *S. xylosus* PSM peptides^1^.

PSM type	Strain(s)	Amino acid sequence	Molecular Weight (Da)^2^
PSMα	HKUOPL8, ATCC 29971, S170, DMSX03, TMW 2.1602	MSFIIDIIKKIVGLFKGE	2052
PSMα (v1)	C2a, SMQ-121, Strain 2, TMW 2.1324, TMW 2.1523,	MSFIIDIIKKIVGLF **T** G **K**	2024
PSMβ1	HKUOPL8, ATCC 29971, S170, DMSX03, TMW 2.1602	MAEIVEAIGKAVSAGLSHDWATMGVSIAEVLGKGVDFVLGFFK	4452
PSMβ1 (v1)	TMW 2.1324, TMW 2.1523, C2a, SMQ-121, Strain 2	M **SG** IVEAIG **N** AV **N** AGL **A** HDWATMG **L** SIAEVLGKGVDF **I** LGFFK	4421
PSMβ2	HKUOPL8, S170, TMW 2.1602, DMSX03, SMQ121, ATCC 29971	MAGLFDAIKETVQAGIAGDGAKLGTSIVSIVENGVGLVSKLFGF	4382
PSMβ2 (v1)	TMW 2.1523	MAGLFDAIKETVQAGIAGDG **V** KLGTSIVSIVENGVGLVSKLFGF	4410
PSMβ2 (v2)	C2a, TMW 2.1324,	MAGLF **E** AIK **N** TVQAGIAGDGAKLGTSIVSIVENGVGLVSKLFGF	4381
PSMβ3	HKUOPL8, S170, TMW 2.1602, DMSX03, ATCC 29971^3^	MTKLAEAIANTVEAAKAGNGADLGSSIVDIVSSGASLVGKLFGL	4248
PSMβ3 (v1)	TMW 2.1324, TMW 2.1523, C2a, SMQ-121	MTKLAEAIANTVEAAK **S** GNGADLGSSIVDIVSSGASLVGKLFGL	4265
PSMβ4	HKUOPL8, S170, TMW 2.1602, DMSX03, ATCC 29971^3^	MAGLFDAIKETVQAGIAGDGAKLGTSIVNIVENGVGLVSKLFGF	4409
PSMβ4 (v1)	SMQ121	MAGLFDAIKETVQAGIAGDGAKLGTSIV **S** IVENGV **A** L **A** SKLFGF	4368
PSMβ5	HKUOPL8, S170, TMW 2.1602, DMSX03	MTKLAEAIANTVEAAKSGSGADLGSSIVDIVSSGASLVGKLFGL	4238
PSMβ5 (v1)	SMQ121	MTKLAEAIANTVEAAKSG **N** GADLGSSIVDIVSSGASLVGKLFGL	4265
PSMβ5 (v2)	ATCC 29971	MTKLAEAIANTVEAAK **S** G **N** GADLGSSIVDIVSSGASLVGKLFGL	4238
PSMβ6	HKUOPL8, S170, TMW 2.1602, SMQ121, ATCC 29971	MEGLFEAIKNTVQAGVAGDGAKLGTSIVSIVENGVALASKLFGF	4411
PSMβ6 (v1)	DMSX03	MEGLFEAIKNTVQAGVAGDGAKLG **I** SIVSIVENGVALASKLFGF	4423
PSMβ6 (v2)	C2a	MEGLFEAIK **S** TVQAG **I** AGDGAKLGTSIVSIVENGVALASKLFGF	4398
PSMβ6 (v3)	TMW 2.1324	MEGLFEAIKNTVQAG **I** AGD **D** AKLGTSIVSIVENGVAL **V** SKLFGF	4511
PSMβ6 (v4)	TMW 2.1523	MEGLFEAIKNTVQAGVAGDGAKLGTSIVSIV **G** NGVALASKLFGF	4339
PSMβ6 (v5)	Strain 2	MEGLFEAIKNTVQAGIAGDGAKLGTSIVSIVENGV **G** L **V** SKLFGF	4439

^1^Amino acid sequences of *S. xylosus PSMs* determined by N-terminal sequencing and with annotated open reading frames in the ten available *S. xylosus* genomes. v, variant (followed by number corresponding to number of variants). Amino acid changes in variants are denoted by underline and bold font. ^2^Note naturally occurring *PSMs* have N-formylated N-terminal methionine residues as they are exported without a leader peptide; thus 28 Da need to be added to the shown theoretical masses. ^3^In ATCC 299771, *psm*β3 (WT) and *psm*β4 (WT) are found in a separate locus next to gene DG061_RS12915.

As for β-type peptides, we found a gene encoding a peptide whose calculated mass exactly corresponded to that of peak 3. We named this peptide PSMβ1 of *S. xylosus* (**Figure 2B**). According to genome analysis, this peptide also can occur as a peptide variant. Furthermore, we found a locus with considerable strain-to-strain variation and apparent gene duplication, encoding maximally five copies of a PSMβ peptide with moderate amino acid sequence variations (**Figure 2C**). Only in one strain, a second locus carrying additional *psm*β genes was found at a different genomic location (**Figure 2D**). We named the corresponding encoded peptides PSMβ2 to PSMβ6 of *S. xylosus*. Re-examining our RP-HPLC/ESI-MS data, we found traces of signals corresponding to the expected masses of some of those peptides in the PSM elution range.

Altogether, our analysis revealed that *S. xylosus* produces a variety of PSM peptides with particularly pronounced variability as indicated by genome analysis. However, many of these peptides only appear to be produced at very low levels, and PSMα or its variant PSMα (v1) and PSMβ1 are the main PSMs produced by *S. xylosus* isolates. There appear to be two main clades of strains, one of which – to which most of our clinical isolates belong – produce PSMα and PSMβ1, while another clade predominantly produces a variant of PSMα, PSMα (v1), and barely PSMβ peptides. Complete absence of PSMs, as observed in one of our clinical isolates and one standard strain, is often found also in *S. aureus* where it is linked to mutation in the Agr quorum-sensing system, which strictly controls PSM production (45, 46). It is likely that this is also the case in the *S. xylosus* strains that do not produce PSMs. Lastly, one defining feature of *S. xylosus* is that it does not produce δ-toxin, a member of the PSM class produced in large amounts in other staphylococci, including *S. aureus* and *S. epidermidis*, for example (21, 47). This absence of a δ-toxin-like peptide in *S. xylosus* is in accordance with no obvious open reading frame being found within the apparent RNAIII gene of *S. xylosus*, where δ-toxin is usually encoded.

### Phenotypic characterization of the two most abundant PSM peptides produced by clinical *S. xylosus* isolates

We focused on the two most abundant PSMs that are produced by the clinical isolates, PSMα and PSMβ1, and characterized their cytotoxic capacities toward human and mouse erythrocytes and neutrophils, as well as their pro-inflammatory capacities by determining calcium flux in human and mouse neutrophils. Calcium flux is the most commonly used readout for the pro-inflammatory capacity of PSMs (40, 48, 49).

**Figure 3:**
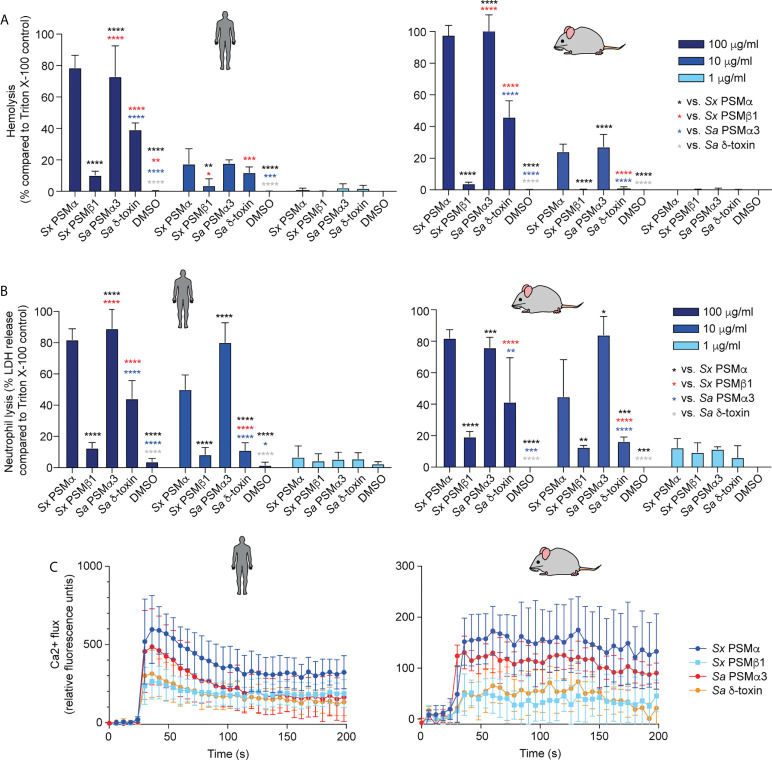
Cytolytic and pro-inflammatory capacities of main *S. xylosus* PSMs. Capacity of synthetic PSM peptides representative of predominant *S. xylosus* PSM species to lyse **(A)** human and mouse erythrocytes, **(B)** Human and mouse neutrophils and **(C)** induce calcium flux in human and mouse neutrophils. Synthetic *S. aureus* PSMα3 and δ-toxin were measured for comparison. For hemolysis and lactate dehydrogenase (LDH) release assays, triplicate measurements were performed with whole heparinized blood and purified neutrophils obtained from three donors, respectively. For calcium flux assays, duplicate measurements were performed from two donors and PSMs were added at a final concentration of 100 nM. Statistical analysis is by 2-way ANOVA with Tukey’s post-tests. *, p<0.05; **, p < 0.01; ***, p < 0.001; ****, p < 0.0001; Error bars show the mean ± SD. Mouse and human schemes are from openclipart.org.

PSMα of *S. xylosus* had strong cytolytic activity toward erythrocytes and neutrophils from humans and mice, at a level comparable to that of *S. aureus* PSMα3, a PSM known to have pronounced cytolytic activity (40) (**Figures 3A, B**). PSMβ1 had low cytolytic activity, as described generally for β-type PSMs of other staphylococci (40, 47, 50). The cytolytic potency of *S. aureus* δ-toxin, an α-type PSM, was higher than that of PSMβ1, but it did not reach the level of activity exerted by the strongly cytolytic *S. xylosus* PSMα or *S. aureus* PSMα3. Notably, there was no apparent species specificity of these effects.

In contrast to the cytolytic activity of PSMs, their pro-inflammatory activity is receptor-dependent (48); thus, these two activities of PSMs are known not to be correlated. PSMα of *S. xylosus* showed the most pronounced pro-inflammatory activity as measured by calcium flux in neutrophils, followed by PSMα3 of *S. aureus*, and PSMβ1 of *S. xylosus* and *S. aureus* δ-toxin with similarly low activity, again with no apparent species specificity (**Figure 3C**).

These data showed that *S. xylosus* produces PSMs with pronounced cytolytic and pro-inflammatory activity, most notably PSMα, but there is no apparent species specificity of their effects.

### Generation and analysis of isogenic *psm* mutants in *S. xylosus*

**Figure 4:**
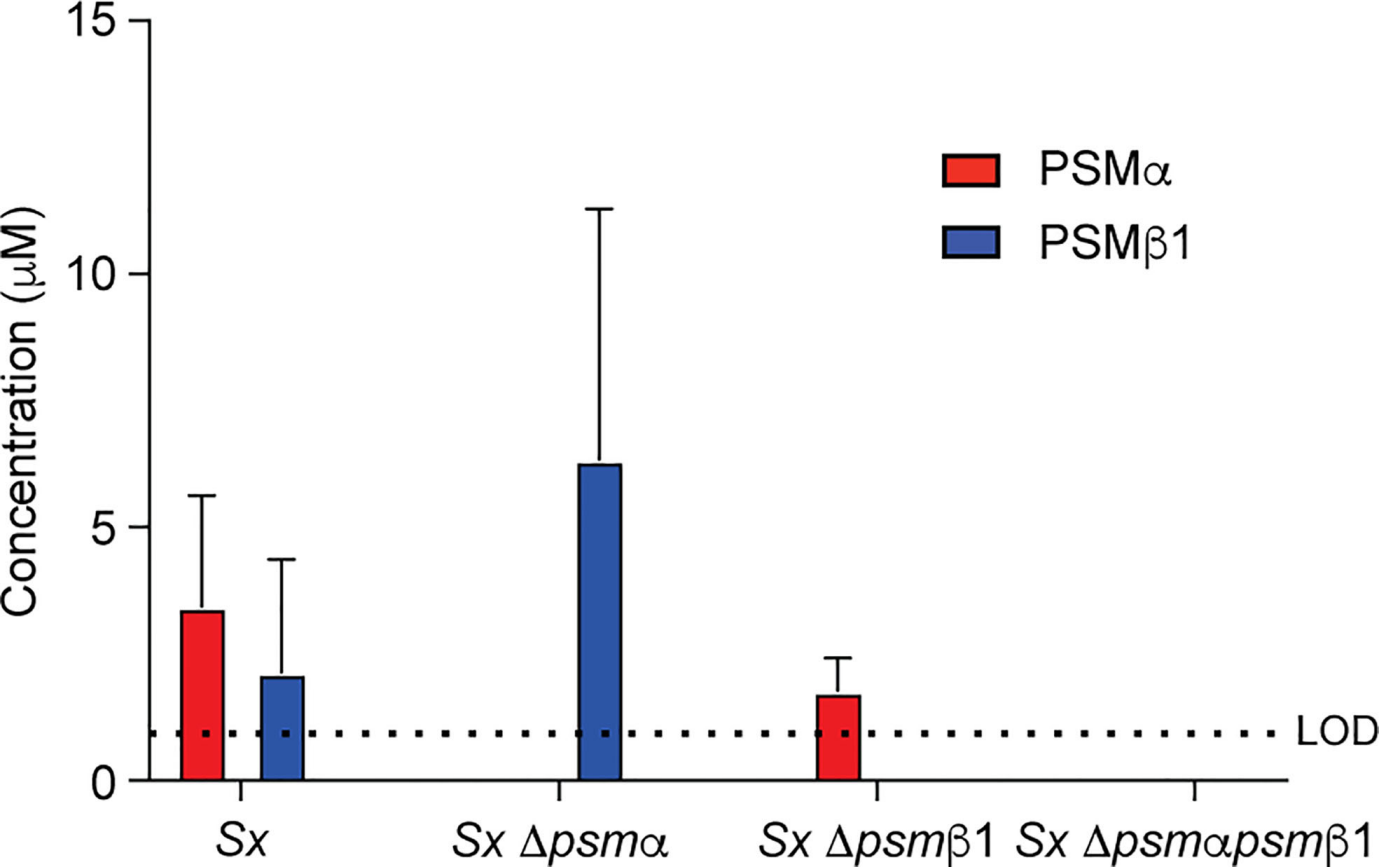
Construction of isogenic *S. xylosus psm* deletion and complementation strains. Analysis of PSM production pattern by RP-HPLC/ESI-MS of culture filtrates collected from constructed isogenic *psm* mutants of strain *S. xylosus* AG14.

Focusing again on PSMα and PSMβ1 as the PSMs with the strongest expression in most of the analyzed *S. xylosus* clinical isolates, we produced single and combined isogenic deletion mutants in the *psm*α and *psm*β1 loci of isolate AG14 as an isolate with high expression of these PSMs. PSM production analysis of the constructed Δ*psm*α, Δ*psm*β1, and Δ*psm*α*psm*β1 mutants showed the expected expression pattern (**Figure 4**). Having established the absence of species specificity of *S. xylosus* PSM activities, we used human erythrocytes and neutrophils for these experiments.

**Figure 5:**
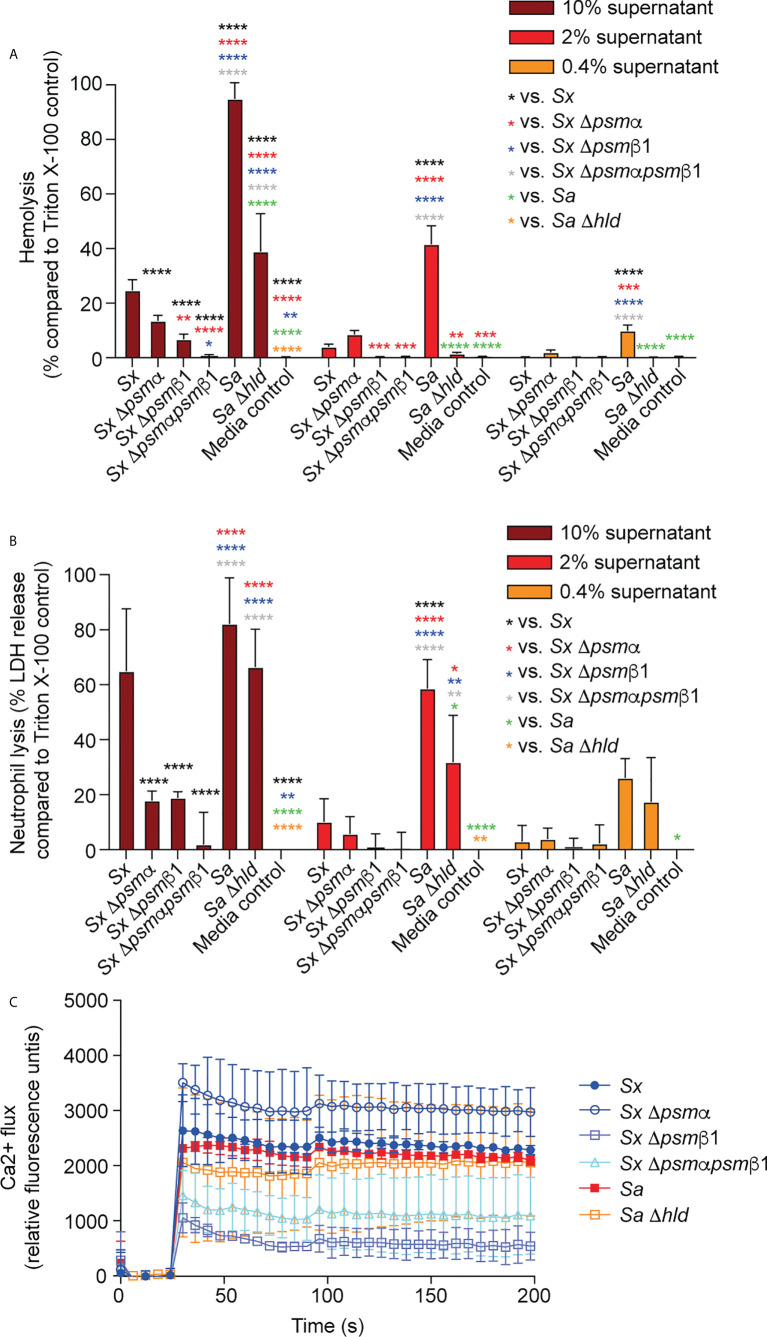
Cytolytic and pro-inflammatory capacities of *S. xylosus psm* deletion strains. Capacity of stationary-phase (16-h) culture filtrates from *S. xylosus* AG14 and isogenic *psm* mutants to induce lysis of **(A)** human erythrocytes, **(B)** human neutrophils and **(C)** induce calcium flux in human neutrophils. Culture filtrates from *S. aureus* LAC and its isogenic δ-toxin mutant were used as comparison. For hemolysis and lactate dehydrogenase (LDH) release assays, triplicate measurements were performed using whole heparinized blood and purified neutrophils obtained from three donors, respectively. For calcium flux assays, single measurements were performed from two donors. Statistical analysis is by 2-way ANOVA with Tukey’s post-tests. *, p<0.05; **, p < 0.01; ***, p < 0.001; ****, p < 0.0001; Error bars show the mean ± SD.

PSMα and PSMβ1 both contributed considerably to cytolysis of erythrocytes and neutrophils in cell-free supernatants of harvested *S. xylosus* cultures (**Figures 5A, B**). Importantly, we could not detect any cytolytic activity when the genes for both these PSMs were deleted, emphasizing that they account for virtually the entire cytolytic activity of *S. xylosus* AG14, and by extrapolation based on PSM production patterns (see **Figure 1B**), of clinical *S. xylosus* strains in general. *S. aureus* δ-toxin was only responsible for part of the cytolytic activity of *S. aureus*, which is in accordance with previous findings (40) and to be expected by the fact that several additional cytolytic PSMs, as well as other cytolysins, are produced by *S. aureus* (51). Of note, as a likely result of the plethora of cytolysins produced by *S. aureus*, lytic activity toward both erythrocytes and neutrophils was considerably more pronounced in *S. aureus* than *S. xylosus*. Contrastingly, the capacity to induce calcium flux of these two species was about the same, with neither the δ-toxin nor PSMα of *S. xylosus* having a significant impact, while PSMβ1 appeared to be responsible for most of the pro-inflammatory effect of *S. xylosus *(**Figures 5C**).

### Mast cell degranulation

While PSMs have been reported to represent important virulence determinants in a series of different *S. aureus* infection types, including skin (40, 52), blood (40), and bone (53) infections, of particular interest for the present investigation is their impact on atopic dermatitis. This is because the isolates we examined are from mice affected with dermatitis-like symptoms. Furthermore, there is discussion in the literature about whether mast cell degranulation as a main mechanism underlying AD pathogenesis is solely impacted by δ-toxin (20) or whether other PSMs also affect AD (23). We therefore examined the impact of *S. xylosus* PSMs on mast cell degranulation and also measured LDH release to determine whether degranulation is linked to cell lysis.

**Figure 6:**
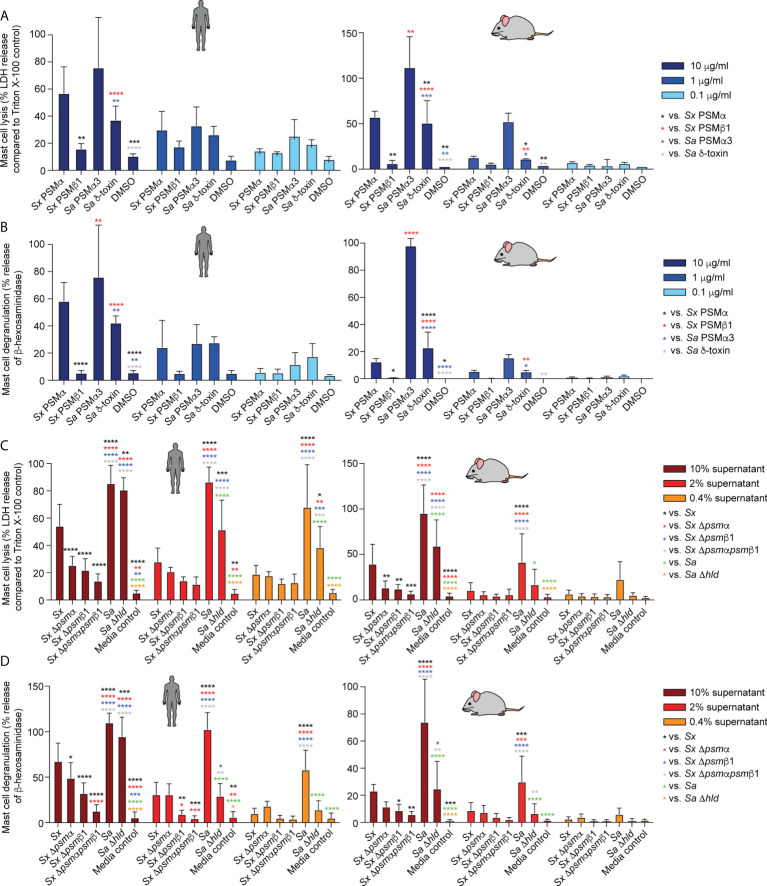
Mast cell degranulation by *S. xylosus* PSMs. Human and mouse mast cell degranulation and lysis by pure PSM peptides **(A, B)** and culture filtrates **(C, D)** of *S. xylosus* AG14 WT and isogenic *psm* mutants is shown. Statistical analysis is by 1-way ANOVA with Tukey’s post-tests. *, p<0.05; **, p < 0.01; ***, p < 0.001; ****, p < 0.0001; Error bars show the mean ± SD. Mouse and human schemes are from openclipart.org.

*S. xylosus* PSMα and PSMα3 of *S. aureus* caused strong, δ-toxin moderate, and *S. xylosus* PSMβ1 only minor mast cell lysis and degranulation (**Figures 6A, B**). Notably, lysis and degranulation by different PSMs were almost identical in degree and pattern. We also again included mouse mast cells in this experiment to verify the absence of species specificity on mast cells as a key cell type promoting AD symptoms. There were virtually no differences in the results obtained with human versus mouse mast cells.

As for the situation in the natural strain background, *S. aureus* supernatant caused considerably stronger lysis and degranulation than *S. xylosus* supernatant (**Figures 6C, D**). The δ-toxin had a strong contribution to mast cell degranulation and lysis, although there was still considerable potency of *S. aureus* supernatant to lyse mast cells and cause degranulation by *S. aureus* supernatant without δ-toxin. Most of *S. xylosus*’ capacity to lyse mast cells and cause degranulation was due to production of PSMα and PSMβ1, with production of both PSMs contributing to those effects. Again, experiments with mouse and human mast cells yielded similar results. As in our experiments with pure PSM peptides, the degree of lysis and degranulation generally was strongly correlated.

### AD model and protein-fragment complementation assay

**Figure 7:**
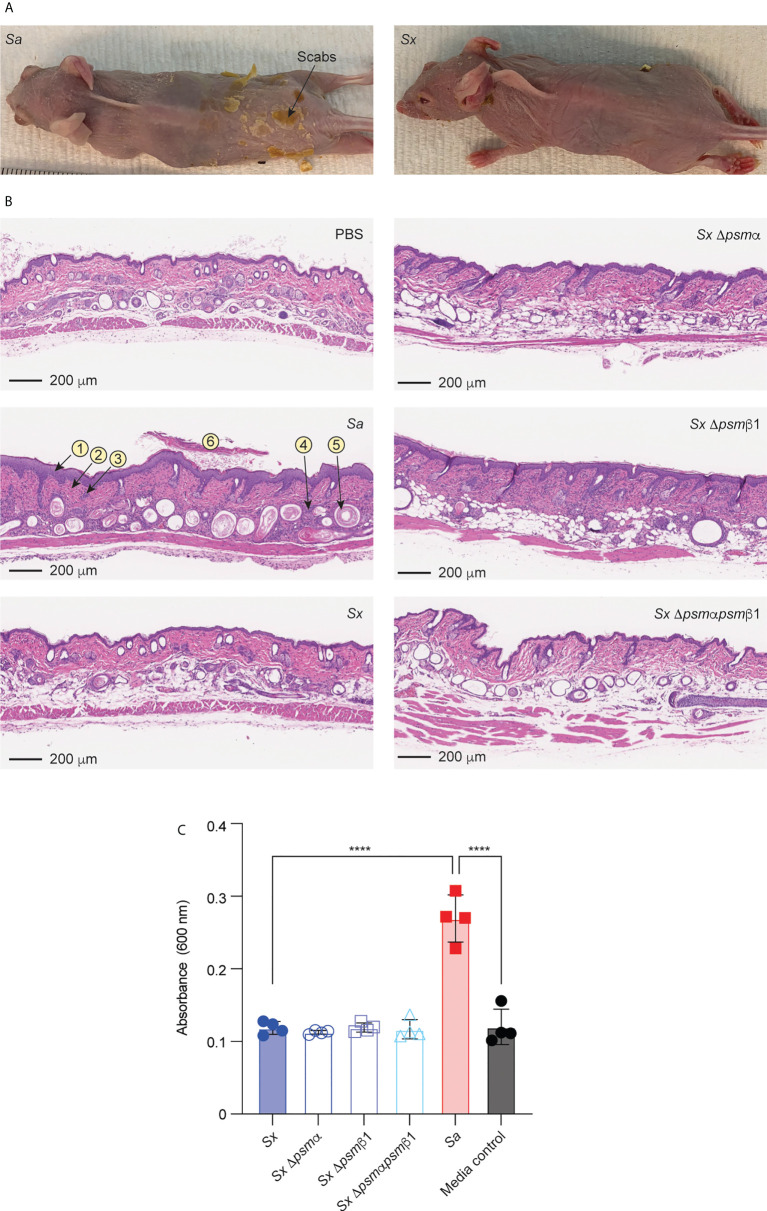
Mouse model of AD and in-vivo mast cell degranulation. **(A)** Representative pictures of mice infected on the skin with *S. aureus* LAC (*Sa*) and *S. xylosus* AG14 (*Sx*). **(B)** Histological evaluation of mice infected with *S. aureus* LAC, *S. xylosus* AG14 or isogenic *S. xylosus psm* mutants. See **Table 2** for scores. Signs of inflammation and infection are marked in the *S. aureus* picture: 1, diffuse hyperplasia; 2, dermal inflammation; 3, focal hyperplasia; 4, follicular inflammation; 5, follicular dilation; 6, hyperkeratosis. These are absent or much less pronounced in all other pictures. **(C)** Mast cell degranulation *in vivo* as determined by a Protein-fragment complementation assay (PCA). Measurements were performed from both ears from two mice after injection with culture filtrates. Statistical analysis is by 1-way ANOVA with Tukey’s post-tests. ****, p < 0.0001; Error bars show the mean ± SD.

We employed an established model of AD in wild-type mice (20, 54, 55) to determine whether the results obtained *in vitro* with mast cells translate to *in vivo* pathogenicity. In accordance with the stronger degree of mast cell degranulation we had observed to be exerted by *S. aureus*, *S. aureus* caused visible scabbing on mouse skin, while *S. xylosus* did not (**Figure 7A**). Similarly, upon histological examination of infected skin tissue, *S. aureus* was found to cause, in all or some mice, follicular inflammation, dilated follicles, focal hyperplasia, diffuse hyperplasia, scabs, ulcers, dermal inflammation, and influx of neutrophils and lymphocytes (**Figure 7B**, **Table 2**). *S. xylosus* only caused these phenotypes to a very minor degree, and there was no difference to the degree of pathogenicity seen with *S. xylosus psm* mutants (**Figure 7B**, **Table 2**). Further confirming these results, in a protein-fragment complementation assay, which we performed to estimate the degree of *in vivo* mast cell degranulation, we observed significantly increased readings in mice injected with culture filtrates from *S. aureus*, but not with *S. xylosus* or its isogenic *psm* deletion mutants (**Figure 7C**).

**Table 2:** Histological evaluation of skin from colonized mice in the atopic dermatitis animal model.

Treatment	Mouse	General score	Follicular inflammation	Dilated follicles	Focal hyperplasia	Diffuse hyperplasia	Scabs	Ulcers	Dermal inflammation	Inflammatory cell types
PBS	1	0	0	0	0	0	0	0	0	N/A
	2	0	0	1	0	0	0	0	0	N/A
	3	0	1	0	0	0	0	0	0	N/A
*S. aureus*	1	3	2	1	0	3	0	0	2	Neu
	2	3	0	4	0	3	3	3	3	Neu, Lymph^1^
	3	3	2	3	4	3	2	2	2	Neu, Lymph
*S. xylosus*	1	2	2	3	2	0	0	0	1	Neu, Lymph
	2	2	2	2	1	0	0	0	1	Neu, Lymph
	3	2	2	2	2	0	0	0	1	Neu, Lymph
*S. xylosus* Δ*psm*α	1	2	0	2	0	2	0	0	1	Neu, Lymph
	2	2	2	2	3	1	0	0	1	Neu, Lymph
	3	2	2	2	0	2	0	0	2	Neu, Lymph
*S. xylosus* Δ*psm*β1	1	2	3	2	2	0	0	0	1	Neu, Lymph
	2	2	1	2	0	2	0	0	3	Neu, Lymph
	3	2	0	2	2	2	0	0	1	Lymph
*S. xylosus psm*α*psm*β1	1	2	1	2	2	0	0	0	1	Lymph
	2	2	2	2	0	2	0	0	1	Neu, Lymph
	3	2	1	3	2	0	0	1	1	Neu, Lymph

^1^Neu, Neutrophils; Lymph, lymphocytes.

## Discussion

In the present study, we identified and characterized PSM peptides of *S. xylosus*, a member of the genus *Staphylococcus* that has been reported to cause AD-like symptoms in mice (27–31). Our study was prompted by previous reports identifying members of the PSM family as virulence factors of the human pathogen *S. aureus* where they promote many infection types including AD (14, 20, 22, 40, 53). Most notably, the δ-toxin of *S. aureus* has been shown to cause AD-associated virulence phenotypes, such as mast cell degranulation, and AD symptoms in mouse models of AD (20). However, other studies have also implied other PSMs of *S. aureus* in mast cell degranulation (23). We thus performed this study not only to characterize what we assumed to be likely major virulence determinants of *S. xylosus*, because non-*S. aureus* staphylococci rarely produce toxins other than PSMs (56, 57), but also to gain insight into the role of δ-toxin versus other PSMs in causing AD.

We found that *S. xylosus* produces three main PSMs, with some strains producing one α- and one β-type PSM, and others mainly one α-type PSM, which is a variant of that found in the other strains. Importantly, no *S. xylosus* strain produced δ-toxin or a δ-toxin homologue. Genome analysis revealed the presence of further loci encoding β-type PSMs, but these genes are apparently only expressed in minor amounts. *S. xylosus* as a species overall showed pronounced heterogeneity regarding PSM production and amino sequence variations, while in other species that we have previously investigated, variants are rarer (21).

In accordance with what we know generally about PSMs (21, 22), the main *S. xylosus* PSMs promoted cytolysis of erythrocytes and neutrophils and calcium flux in neutrophils. The cytolytic activities of the two main *S. xylosus* PSMs were consistent in degree toward the different cell types, while the relative degree of pro-inflammatory activities differed from the cytolytic activity pattern, as expected from the fact that the cytolytic activities are not receptor-dependent while the pro-inflammatory activities are (48). Absence of production of both main *S. xylosus* PSMs in a constructed double mutant led to virtually complete reduction of those capacities. As for the cytolytic capacities this is expected from the general absence of cytolysins other than PSMs in these species. Like in *S. aureus*, where PSMs are by far the most pro-inflammatory secreted components (36), pro-inflammatory capacity of *S. xylosus* was also mainly due to PSMs.

Mast cell degranulation is a hallmark and key disease phenotype of AD (17). We therefore measured mast cell degranulation to estimate the potency of *S. xylosus* PSMs to contribute to AD development. Intriguingly, the potency of *S. xylosus* PSMα, as well as of *S. aureus* PSMα3, exceeded that of *S. aureus* δ-toxin, the main PSM previously associated with AD. Furthermore, the lytic activities toward mast cells correlated well with those toward erythrocytes and neutrophils as well as the abilities to promote mast cell degranulation. Nevertheless, cytolytic and mast cell degranulation potency of *S. aureus* supernatant was much higher than that of *S. xylosus* and to a large part due to δ-toxin.

The mechanism by which δ-toxin contributes to AD development has remained unknown, Nakamura et al. reported that interaction with the PSM receptor FPR2, which underlies the pro-inflammatory capacities of PSMs (48), is not involved (20). These authors also reported that sublytic concentrations of δ-toxin can cause mast cell degranulation, but that this is enhanced in the presence of anti-DNP IgE (20), suggesting that δ-toxin can trigger mast cell degranulation by an FcϵRI-dependent mechanism by engaging with the IgE-FcϵRI complex. On the other hand, in this and another study a correlation was found between mast cell degranulation and cytolytic potency of highly cytolytic α-type PSMs (20, 23), which is in accordance with our findings that show strong correlation of mast cell degranulation with general cytolytic capacity. This may indicate that lytic capacity determines δ-toxin’s degranulating activity on mast cells. While our findings imply a potential contribution of a mechanism that is based only on cytolysis, we planned our degranulation experiments always in the presence of IgE. Therefore, we are unable to comment further as to which mechanism has more significance. Notably, our findings indicate that the higher abundance of δ-toxin in *S. aureus* supernatants as compared to other PSMs (40, 47), rather than an exceptional potency to trigger mast cell degranulation, explain the increased corresponding potency of *S. aureus* as compared to *S. xylosus*.

While our results on mast cell degranulation indicate potency of *S. xylosus* to promote AD, albeit at a lower extent than *S. aureus*, we were not able to detect significant AD-like symptoms in an AD model using wild-type mice in which, contrastingly, *S. aureus* showed a clear impact. That we also could not detect an impact of *psm* genes of *S. xylosus* is likely due to already only very minor pathogenicity readouts we detected for *S. xylosus* wild-type. Given that AD-like symptoms in mice were reported exclusively in mice that were genetically or otherwise predisposed (27–31), similar to the mice from which the *S. xylosus* strains were isolated for this study, we believe that – in contrast to *S. aureus* – *S. xylosus* only has the potency to promote AD in the presence of other predisposing factors.

In conclusion, the findings of our study, which identified and characterized PSMs of *S. xylosus*, further substantiate the notion of widespread production in the genus *Staphylococcus* of cytolytic and pro-inflammatory peptides of the PSM family and underlines the previously emphasized species specificity of PSM production (21, 58). Furthermore, our findings have important implications for our understanding of PSM involvement in AD, since they suggest that the previously noted exceptional corresponding capacity of δ-toxin (20) is not due to specific characteristics of that peptide as compared to other PSMs, but its production levels. Because of that situation, a species like *S. xylosus* with absence of δ-toxin or other highly produced PSMs likely requires predisposing host factors for a PSM-mediated impact on AD development. Further exploration will be necessary to more completely understand the role of δ-toxin and other PSMs in AD development, such as to address questions that arise from the fact that in *S. epidermidis* for example, a δ-toxin homologue that is very similar to that of *S. aureus* is produced in high amounts (47); yet – despite some reports on the potential of *S. epidermidis* factors to trigger AD phenotypes (59) and *S. epidermidis* association with AD flares (18) - this species has not been associated with AD as clearly as *S. aureus*. Of potential importance in that regard, *S. epidermidis* does not produce high amounts of strongly lytic PSMs (47, 57). Thus, we speculate that the combined cytolytic potency of secreted PSMs determines the degree to which a given species or strain triggers mast cell degranulation and thereby, AD, a notion that will need to be substantiated by investigation with large numbers of different species and strains.

## Data availability statement

The original contributions presented in the study are included in the article/**Supplementary Material**. Further inquiries can be directed to the corresponding authors.

## Ethics statement

The animal study was reviewed and approved by Division of Intramural Research Animal Care and Use Committee (DIR ACUC) of the National Institute of Allergy and Infectious Diseases (NIAID).

## Author contributions

AG and WE collected and provided *S. xylosus* skin isolates from mice. HJ, YZ, and MO performed RP-HPLC/ESI-MS analyses for identification and detection of PSMs. RH, GC and MO performed bioinformatical analyses. AV and JB created *S. xylosus* isogenic *psm* deletion and complementation strains. KR, RH, RL, TN, GC, and JB performed cellular experiments. KR, RH, RL, GC, and JB performed animal experiments. KR, GC and MO analyzed data. GC and MO designed the study. GC and MO supervised experiments. GC and MO wrote the paper. All authors contributed to the article and approved the submitted version.

## Funding

This study was supported by the Intramural Research Program of the National Institute of Allergy and Infectious Diseases (NIAID), Laboratory of Bacteriology (project number ZIA AI000904 to MO) and the Comparative Medicine Branch, U.S. National Institutes of Health (NIH).

## Acknowledgments

The authors thank Dr. Dean Metcalfe (Laboratory of Allergic Diseases, NIAID, NIH) for the kind gift of LAD2 cells and Dr. Jerrold Ward for thehistopathological evaluation of skin samples.

## Conflict of interest

The authors declare that the research was conducted in the absence of any commercial or financial relationships that could be construed as a potential conflict of interest.

## Publisher’s note

All claims expressed in this article are solely those of the authors and do not necessarily represent those of their affiliated organizations, or those of the publisher, the editors and the reviewers. Any product that may be evaluated in this article, or claim that may be made by its manufacturer, is not guaranteed or endorsed by the publisher.
