# Supplementary material for: Identification and characterization of the pathogenic potential of phenol-soluble modulin toxins in the mouse commensal Staphylococcus xylosus
Source: Front Immunol. 2022 Sep 15;13:999201. doi: 10.3389/fimmu.2022.999201 (PMC9520458; doi:10.3389/fimmu.2022.999201)
Supplement: Supplementary file 1 [file DataSheet_1.pdf]

Identification and characterization of the pathogenic potential of phenol-soluble modulin toxins in the mouse commensal *Staphylococcus xylosus*

Kunal Reshamwala, Gordon Y. C. Cheung, Roger C. Hsieh, Ryan Liu, Hwang-Soo Joo, Yue Zheng, Justin S. Bae, Thuan H. Nguyen, Amer E. Villaruz, Alfonso S. Gozalo, William R. Elkins, Michael Otto

Supplementary Material

Supplementary Table S1. List of bacterial isolates used in this study.

Bacterial and viral strains	Relevant genotype and property	Source
Escherichia coli DH5α	*endA*1 *recA*1 *gyrA*96 *thi-1* *hsdR17*($r_κ^-$ $m_κ^+$) *relA*1 *supE*44 (*lacZYA-argF*) U169 F-80d*lacZ*M15 *deoR phoA*	Invitrogen
Staphylococcus aureus RN4220	Derived from NCTC8325-4; r^- m^+	(1)
Staphylococcus aureus LAC (*Sa*)*	Epidemic U.S. CA-MRSA clone, strain LAC, ST8, CC8	(2)
Staphylococcus aureus LAC Δ*hld* (*Sa* Δ*hld*)	The *hld* start codon altered to abolish translation	(3)
Staphylococcus xylosus C2a	Derived from DSM20267: cured of endogenous plasmid pSX267	(4)
Staphylococcus xylosus ATCC 29966	Clinical isolate from human skin	ATCC
Staphylococcus xylosus isolates AG8, AG10, AG11, AG12, AG14 (*Sx*), AG15	Clinical isolate wild-type strain from a mouse background (C57BL/6 WT or related strain) with spontaneous ulcerative dermatitis (NIAID mouse breeding facility, NIH)	This study
Staphylococcus xylosus AG14 Δ*psm*α (*Sx* Δ*psm*α)	Isogenic markerless *psm*α deletion mutant of *S. xylosus* AG14.	This study
Staphylococcus xylosus AG14 Δ*psm*β1 (*Sx* Δ*psm*β1)	Isogenic markerless *psm*β1 deletion mutant of *S. xylosus* AG14.	This study
Staphylococcus xylosus AG14 Δ*psm*α*psm*β1 (*Sx* Δ*psm*α*psm*β1)	Isogenic markerless *psm*α and *psm*β1 deletion mutant of *S. xylosus* AG14.	This study
Staphylococcus carnosus TM300		(5)
Staphylococcus aureus PS187Δ*sauUSI* Δ*hsdR* (PS187ΔΔ)	*S. aureus* strain PS187 deficient in type IV and type I restriction modification system	(6)
Φ187	Siphoviridae, Serogroup L	Gift from A. Peschel

* Names in parentheses denote abbreviated strain name used in the manuscript

Supplementary Table S2. Sequences of the primers used for generation of isogenic *psm* deletion mutants in *S. xylosus*

Nucleotide name	Primer used	Purpose
Xylpsmα1	GGTGTATGGCAAGTGACTCCAATTAAACA CGG	Allelic replacement of *psm*α
Xylpsmα1 (Reverse)	CTAGAAATTATTCGAGGCCGAGACATTC	Allelic replacement of *psm*α
Xylpsmα2	CGAATAATTTCTAGGTATCTCACCTCGCTT TGTTTTGTTAAG	Allelic replacement of *psm*α
Xylpsmα2 (Reverse)	CACCTACTTGCGATTGCACATACCATCG	Allelic replacement of *psm*α
Xylpsmβ1	TAATTAACCCAATGTAATCACAGCGTTCG	Allelic replacement of *psm*β1
Xylpsmβ1 (Reverse)	TTTTGAATTTAAAATTAAAACATTTACTTT	Allelic replacement of *psm*β1
Xylpsmβ2	AAAGTAAATGTTTTAATTTTAAATTCAAAA AAACATATGCATTAAAAAGGCCTTTTCTAG A	Allelic replacement of *psm*β1
Xylpsmβ2 (Reverse)	GGAGATTAATGTTGGGTATTGCGGCTGTTC	Allelic replacement of *psm*β1

References

1. Vuong C, Gerke C, Somerville GA, Fischer ER, Otto M. Quorum-sensing control of biofilm factors in *Staphylococcus epidermidis*. J Infect Dis. 2003;188(5):706-18.
2. Centers for Disease C, Prevention. Outbreaks of community-associated methicillin-resistant *Staphylococcus aureus* skin infections--Los Angeles County, California, 2002-2003. MMWR Morb Mortal Wkly Rep. 2003;52(5):88.
3. Wang R, Braughton KR, Kretschmer D, Bach TH, Queck SY, Li M, et al. Identification of novel cytolytic peptides as key virulence determinants for community-associated MRSA. Nat Med. 2007;13(12):1510-4.
4. Gotz F, Zabielski J, Philipson L, Lindberg M. DNA homology between the arsenate resistance plasmid pSX267 from *Staphylococcus xylosus* and the penicillinase plasmid pI258 from *Staphylococcus aureus*. Plasmid. 1983;9(2):126-37.
5. Schleifer KH, Fischer U. Description of a New Species of the Genus *Staphylococcus* - *Staphylococcus carnosus*. Int J Syst Bacteriol. 1982;32(2):153-6.
6. Winstel V, Xia G, Peschel A. Pathways and roles of wall teichoic acid glycosylation in *Staphylococcus aureus*. Int J Med Microbiol. 2014;304(3-4):215-21.
